# A new circular economy approach for integrated production of tomatoes and mushrooms

**DOI:** 10.1016/j.sjbs.2021.12.058

**Published:** 2022-01-03

**Authors:** Vandinelma Oliveira Vieira, Aparecido Almeida Conceição, Joice Raisa Barbosa Cunha, Antony Enis Virginio Machado, Euziclei Gonzaga de Almeida, Eustáquio Souza Dias, Lucas Magalhães Alcantara, Robert Neil Gerard Miller, Félix Gonçalves de Siqueira

**Affiliations:** a Federal University of Mato Grosso, Biotechnology and Biodiversity of the Pro Centro Oeste Network, Cuiabá, Mato Grosso, Brazil; b Federal University of Lavras, Agricultural Microbiology, Lavras, Minas Gerais, Brazil; c Federal University of Tocantins, Guripi, Tocantins, Brazil; d Federal University of Mato Grosso, Institute of Biosciences, Department of Botany and Ecology, Cuiabá, Mato Grosso, Brazil; e Federal University of Lavras, Department of Biology, Lavras, Minas Gerais, Brazil; f University of Guelph, Department of Animal Biosciences, Guelph, Ontario, Canada; g University of Brasilia, Distrito Federal, Brazil; h Embrapa Agroenergy, Brasília, Distrito Federal, Brazil

**Keywords:** Fungiculture, Tomato and mushroom co-cultivation, Green biostimulants, SMS

## Abstract

Spent mushroom Substrate is the by-product generated at the end of the mushroom growing cycle. It can be used in agriculture for different purposes, including seedling production, soil conditioning or application as an organic fertilizer. Tomato is one of the world́s most important crops, requiring considerable care, in terms of both nutrition and disease control. The objective of this study was to investigate the viability of spent mushroom substrate as a nutrient source for tomato seedlings and develop an integrated tomato and mushroom co-production system. For seedling production, different compositions were evaluated with spent mushroom substrate from *Pleurotus ostreatus* or substrate colonized with *Agaricus bisporus*. The parameters evaluated comprised germination rate, seedling quality and physicochemical analysis. A tomato and mushroom integrated production system was developed using a 40-liter pot divided into upper (spent mushroom substrate and soil), middle (spent mushroom substrate from *P. ostreatus*) and lower (gravel) layers. For seedlings production, plants treated with the substrate colonized with *A. bisporus* presented a superior root length (10.1 cm) and aerial part length (6.6 cm). Co-production of tomato and mushrooms was also shown to be viable. In this co-cultivation system between tomato and mushroom, the treatment with the substrate colonized with *A. bisporus* differed from others, with this treatment presenting high yields of tomato (2.35 kg/plant pot) and mushrooms (1.33 kg/plant pot) within the same bucket. With this co-production system, the tomato production time was reduced by 60 days and prolonged continuous mushroom production by 120 days. These findings show a sustainable approach to manage different agroindustrial residues, encouraging the use of these residues for olericulture and fungiculture production.

## 1 Introduction

Spent Mushroom Substrate (SMS) is a by-product of the mushroom industry. During fungal cultivation, colonized biomass undergoes a series of alterations, often considered as a pre-treatment by the fungus, which, together with the mycelial mass and its constituents (glucan, chitin, etc.), result in the production of SMS with a series of characteristics that make it a product with high biotechnological value for development of bioproducts for agriculture, fuels and livestock (Grimm and Wösten, 2018, Hassan et al., 2019, Siwulski et al., 2019).

Mushrooms can be cultivated on varied agroindustry residual green biomasses (RGB). Such versatility enables different activities to be interconnected, such that a co-product generated by one activity can serve as a raw material for a subsequent activity. In this context, a number of co-products from Brazilian agriculture, including cereals, oilseeds, straw and bagasse, can all be applied for mushroom production, which, in turn, can subsequently generate SMS that can be employed as substrate for vegetable and fruit seedling production (Collela et al., 2019). As such, SMS is a co-product with important biotechnological potential, suitable for connecting agricultural systems within the precepts of the circular economy (Grimm et al., 2020, Meyer et al., 2020, Wan Mahari et al., 2020, Zied et al., 2020). The palm oil (*Elaeis guineensis*) agribusiness, for example, generates large amounts of RGB. In 2017, the State of Pará (Brazil) produced approximately 480 thousand tons of oil, together with approximately 1.4 million tons of solid and liquid waste (Marlina et al., 2015). In addition to mushroom production, these biomasses can have numerous applications, including thermal elnergy, second generation fuels, chemicals, biomaterials and seedling production (Abdeltawab and Khattab, 2018, Aziz and Hanafiah, 2020, Madusari, 2018, Mahidin et al., 2020, Triyono et al., 2019).

For the production of vegetable seedlings, alternative substrates can offer potential in both reduction of costs and in the development of sustainable and environmentally friendly growing systems (Kraska et al., 2018). In this context, tomato (*Solanum lycopersicum*) is an excellent model for investigation, given its economic importance and phytosanitary particularities.

In order to improve efficiency in mushroom production, diversify sources of vegetable biomass have been evaluated for the preparation of substrates, mainly according to regional availability (de Siqueira et al., 2011, Gonçalves et al., 2010, Martos, 1932). In this context, palm oil-producing regions, such as the state of Pará in Brazil, offer potential for the implementation of circular economy models, integrating production of oils (palm oil), mushrooms, olericulture (tomatoes) and substrates for vegetable seedlings. The integration of production chains (palm oil culture and fungiculture) has been shown to be possible through the use of by-products or co-products from palm oil industry (Kumla et al., 2020, Triyono et al., 2019).

The employment of substrates that are abundant and efficient for seed germination can reduce costs in the agricultural sector, especially for smallholder producers (Madusari, 2018, Yau and Murphy, 2000). In this context, there has been a significant increase in the use of sustainable and/or environmentally friendly substrates as alternatives to inorganic or peat-based products for the production of greenhouse vegetables.

The aim of this study was to investigate the feasibility of employing colonized mushroom substrates (*Agaricus bisporus*), SMS (*A. bisporus* and *Pleurotus ostreatus*), and palm oil residues as substrates for the production of tomato seedlings. The study demonstrated a new system for integrated mushroom and tomato production in acclimatized greenhouses, as a model of integration of palm oil production, fungiculture and olericulture within the precepts of the circular economy.

## 2 Materials and methods

### 2.1 Biomaterials employed in substrate formulations

Experiments on tomato seedling production and integrated cultivation of tomato and *P. ostreatus* were carried out in a controlled greenhouse facility at Support Center for Energy Cultures (*Núcleo de Apoio a Culturas Energéticas* -NACE), an extension of Embrapa Agroenergy.

The SMS of *P. ostreatus* (SMS-P), SMS of *A. bisporus* (SMS-A) and substrate colonized by *A. bisporus* (CAB) were all acquired from local commercial mushroom producers in the Federal District and the states of São Paulo and Paraná.

For the composting preparation, *P*. *ostreatus* SMS was mixed with grasses (*Brachiaria* and *Andropogon*) and chicken manure in a proportion of 3:2:1 (v/v dried base), moistened at 70%. This compost mixture was piled up and turned over every two days, for a period of 3 weeks. The compost was further manipulated, as follows: (1) drying at room temperature for production of *Pleurotus ostreatus* Phase I compost (SC-1); (2) aeration of SC-1 in a mechanized forced air system (air compressor type) for a period of seven days for preparation of *Pleurotus ostreatus* phase II compost (SC-2). Both composts were crushed (0.5 mm) to obtain a granulometry compatible with the seed germination tray cells. *Agaricus bisporus* was cultivated in a previously composted substrate obtained directly from the mushroom farmer.

The bacterial strain *Pseudomonas putida* KT2440 (B) was selected based on its potential in plant growth promotion (Carrasco et al., 2019, He et al., 2019, Li et al., 2019). The strain was inoculated in Luria Bertani liquid medium and incubated under agitation (180 rpm) at 30 °C for a 7-day period. The culture was then centrifuged, resuspended in sterile distilled water, and adjusted to a concentration of 1.25x10^9^ cells/mL. An aliquot of 1 mL was applied per cell tray containing tomato seeds.

### 2.2 Tomato seedling production treatments

Cherry tomato seedling production (*Solanum lycopersicum* Mill. var. cerasiforme) was conducted under controlled environmental conditions of an average temperature of 30 °C, and micro aspersion irrigation three times a day for 15-minute periods to maintain humidity at 70%. Two seeds were sown per cell in rigid polystyrene trays previously filled with each treatment. After sowing, the seeds were covered with 1 cm of substrate. All experiments were conducted in triplicate.

Several formulations were prepared containing SMS as the main component of the seedling substrate. Across the substrate formulations, eight different treatments were assessed for seedling production that contained the following materials: soil (dystroferric red latosol – sieved (0.45 mm)), substrate colonized with *A. bisporus*, *A. bisporus* SMS, *P. putida*, SC-1 and SC-2. Proportions employed for each treatment are described in Table 1 (T1 to T8). The commercial substrate Carolina Soil Class XVI (Composed of peat, vermiculite, organic waste, class A agro-industrial organic waste and limestone) was included as the control treatment for all experiments. Treatment 1 (T1-Table 1) was further examined for suitability in formulations, following addition of different nutritional conditioners (NCs) in various combinations and proportions (T9 to T12). The NCs employed in formulations were derived from residues from the palm oil agroindustry. Finally, substrate formulation T10 was also further evaluated following the addition of vermiculite (25% and 50%) (T13 to T14 - Table 1).

**Table 1:** Substrate formulations for cherry tomato seedling production.

Treatments	Substrate formulation description	Substrate components (%)
Control	Carolina Soil Commercial Substrate	100
T1	Substrate colonized with *A. bisporus*	100
T2	SMS *A. bisporus*	100
T3	SMS *A. bisporus* + soil	75/25
T4	SMS *P. ostreatu*s + *P. putida*	100
T5	Substrate for *Pleurotus* phase I	100
T6	Substrate for *Pleurotus* phase II	100
T7	SMS *A. bisporus* + SMS *P. ostreatus*/*P. putida* + soil	25/25/50
T8	SMS *A. bisporus* + SMS *P. ostreatus*/*P. putida*	50/50
T9	T1 + Palm kernel cake + palm oil ash + palm oil fiber	50 + (15/15/20)
T10	T1 + Palm kernel cake + palm oil ash + palm oil fruit bunch	50 + (15/15/20)
T11	T1 + Palm kernel cake + palm ash	50 + (25/25)
T12	T1 + Palm kernel cake + palm oil ash + palm oil fiber + palm oil fruit bunch	50 + (15/15/10/10)
T13	T10 + vermiculite	75/25
T14	T10 + vermiculite	50/50

### 2.3 Mineralogical composition

The substrate from treatment T10 (substrate colonized with *A. bisporus +* Palm kernel cake + palm oil ash + palm oil fruit bunch) and the control treatment (commercial substrate) were further analysed in terms of pH, cation-exchange capacity (CEC), humidity (at 60–65 **°**C), percentage of organic Carbon, total organic matter, total Nitrogen, total Phosphorus (P_2_O_5_), Potassium (K_2_O), Zinc, Manganese, Calcium, Magnesium, Sulfur, Boron, Copper, and Iron. All mineralogical analyses were performed by *Nativa Laboratório de Agrícolas LTDA*, on the basis of inductively coupled plasma atomic emission spectroscopy (ICP-OES) (Embrapa, 1997).

### 2.4 Germinative and quality parameters of seedlings

Evaluations relating to germination were conducted from sowing to thinning (20 days). Calculated variables comprised germination (G), germination speed index (GSI), mean germination speed (MGS) and mean germination time (MGT). Analysis of physical quality parameters was performed 30 days after sowing, with focus on root length, aerial length, wet mass, and dry mass according to Collela et al. (2019). For evaluation of variables in the seedling phase, three random plants were collected to compose one experimental unit.

### 2.5 Integrated production of tomatoes and mushrooms (*Pleurotus ostreatus*)

**Fig. 1:**
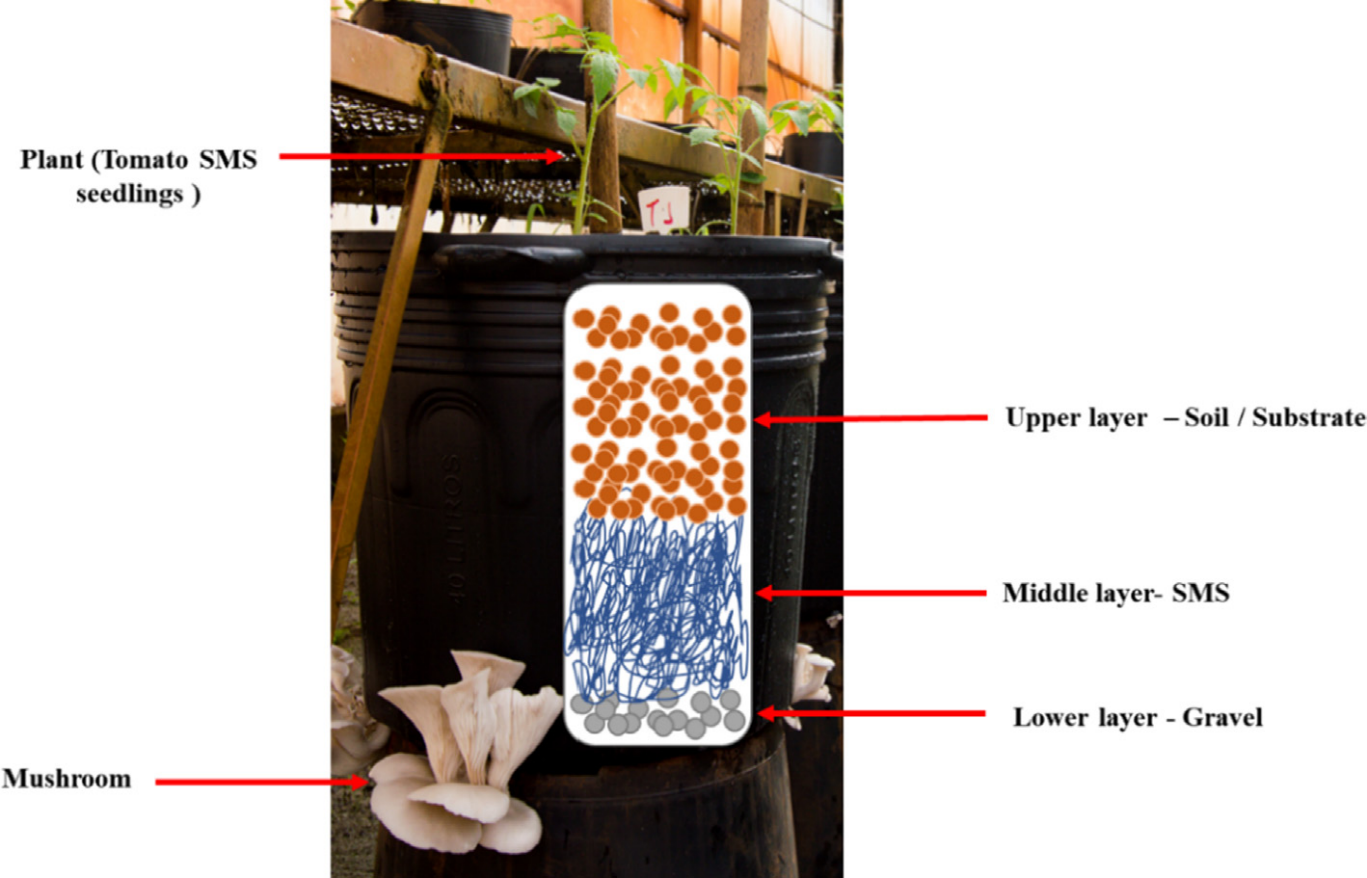
Layering scheme employed in the integrated cherry tomato and mushroom co-cultivation production system, conducted in 40-liter plastic pots inside an acclimatized greenhouse.

For the integrated mushroom and tomato cultivation system, henceforth referred to as co-cultivation, plastic pots with 40 L capacity were filled with three distinct layers (Fig. 1). Since fresh SMS is composed of viable living fungal mycelium and plant biomass with depleted nutrients, what incapacitate mushroom production, it is hypothesized that adding more nutrients to this residue, especially nitrogen, may allow the fungus to produce mushrooms a second time. Thus, the nitrogen present in the soil supplement can provide the necessary nutrients for the fungal fructification reactivation.

**Fig. 2:**
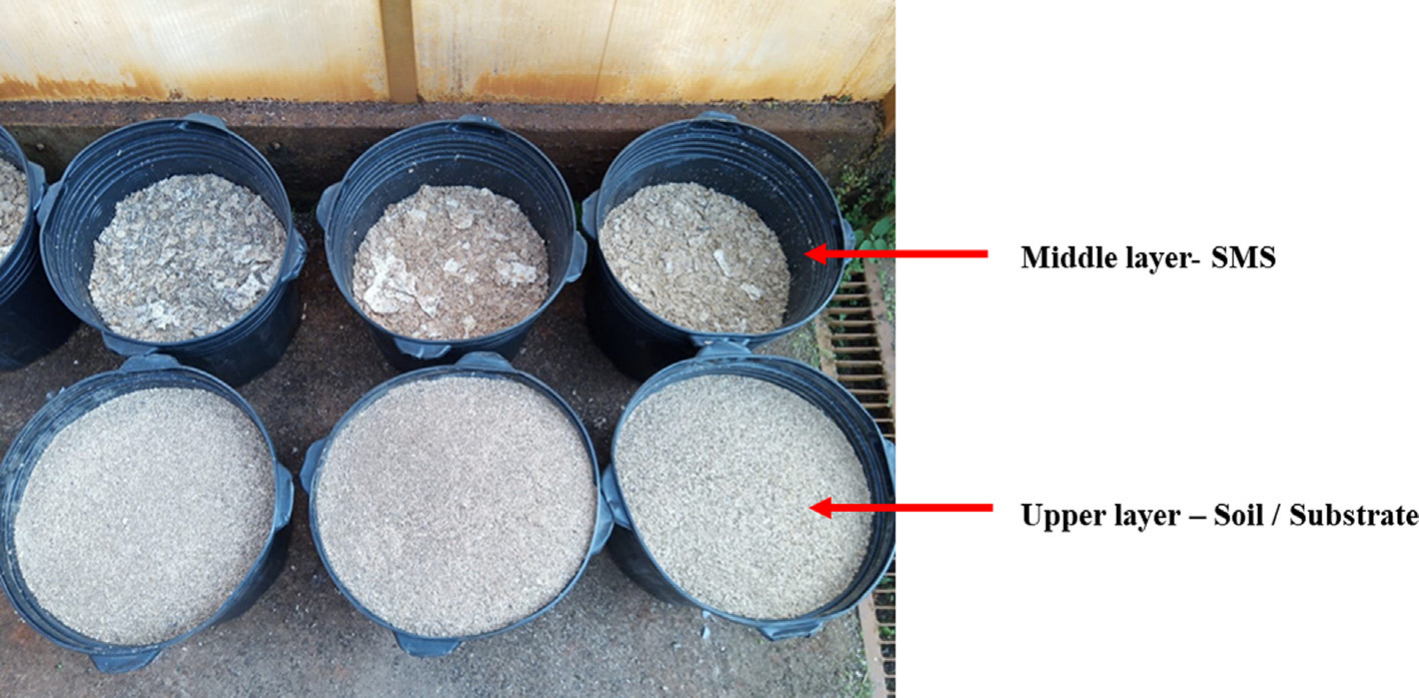
Illustrative picture showing pots filled with a middle layer of SMS and an upper layer with soil mixed with diverse substrates employed in the integrated cherry tomato and mushroom co-cultivation production system.

The co-cultivation experimental design consisted of five treatments with eight repetitions each, varying in terms of the composition of the soil and substrate in the upper layer (layer 3 - Fig. 1, Fig. 2), as described in Table 2. The soil-substrate content used in this layer were mixed proportionally (v/v). The volume of SMS employed in the middle layer (layer 2 - Fig. 1**,** Fig. 2) was also proportional (v/v) to the upper layer volume. The lower layer (layer 1 - Fig. 1) consisted of medium sized gravel, up to approximately 3 cm depth, to enable drainage of excess water or slurry, while also allowing mushroom fruiting. After the pots were assembled with all the layers, tomato seedlings were planted in the upper layer. For this, seedlings originated from the substrate treatment T10 ((Substrate colonized with *A. bisporus +* Palm kernel cake + palm oil ash + palm oil fruit bunch), given the positive results in the previous tests.

**Table 2:** Composition of the upper layer treatments employed in cherry tomato and mushroom (*P. ostreatus* - commercial SMS from two harvests) co-cultivation.

Treatment	Upper layer composition
T1	Soil + Superficial fertilization with commercial fertilizer (5.7 g Ammonium Sulfate, 19 g MAP (Monoammonium Phosphate), 7 g KCl (Potassium Chloride)
T2	50% soil + 25% substrate colonized with *A*. *bisporus* + 12.5% Palm kernel cake + 12.5% Palm fruit bunch fiber
T3	50% soil + 50% substrate for the cultivation of *A. bisporus* (same used in experiment 2).
T4	50% soil + 50% substrate colonized with *A. bisporus*
T5	50% soil + 50% SMS *P. ostreatus*

For each of the treatments, after seedling planting, upper layers were irrigated and maintained in the greenhouse under high humidity (85%) and a temperature range between 25 and 35 °C. Nebulization was conducted twice a day for 5 min periods (at 6:00 a.m. and 6:00p.m.).

After 60 days, the top layer from each treatments, with the exception of T1 (commercial substrate control), were supplemented (nutrient and soil replacement) with 3 L of a mixture containing 60% soil, 20% *P. ostreatus* SMS and 20% chicken manure compost (Plante-Brasil - Super-Adubo®, Composition: pH 8, CTC-C 5.33, C/N Ratio 5, humidity 30%, total P_2_O_5_ 1%, total N 3%, total Mg 1%, H_2_O.K_2_O 2%, total Ca 4%, organic carbon 15% and CTC 80 mmol C/kg). After supplementation, co-cultivation was extended for a further 60 days.

Mushrooms were harvested over 120 days during co-cultivation, across two periods (i) 0–60 days and (ii) 61–120 days. Production was calculated according to the average ratio of quantity (kg) of fresh mushrooms per pot and biological efficiency (EB%) (ratio of fresh mushrooms harvested (grams) per dry substrate (grams)), calculated on the basis of the following formula:

$$\mathrm{EB}\%=\frac{\mathrm{freshweightofmushroom}(g)}{drysubstrateweight\left(g\right)}*100$$

Tomatoes were harvested from the initial stage of ripening (red spots) until ripe (totally red). The yield of the tomato harvest was calculated according to the average of tomatoes (kg) produced per pot.

### 2.6 Statistical analysis

Statistical analysis was performed using the program R, version 3.4.3. A completely randomized design was employed in the experiments, with means compared by the Scott Knott and Tukey test at the 5% significance level, with normality and homogeneity verified on the basis of Shapiro Wilk, Bartlett and Poisson tests.

## 3 Results

### 3.1 Formulations for tomato seedlings

The qualitative parameters for the development of cherry tomato seedlings in different substrates are presented in Table 3. We observed that the commercial control presented parameters superior to those observed in other treatments, indicating the need to improve the formulations T1 to T8. With regard to T1 (substrate colonized with *A. bisporus*), even when germination speed index and germination speed values were inferior to those observed in other treatments, seedling vigor, measured in terms of root length and aerial part length, stood out when comparing with data from other treatments (Table 3). Specifically, T1 presented a germination percentage statistically equal to that of the control (100%), with root length greater than in all other treatments and aerial part length statistically equal to T2 (SMS *A. bisporus*) and T7 (SMS *A. bisporus* + SMS *P. ostreatus*/*P. putida* + soil).

**Table 3:** Germination and physical parameters (root and aerial part length) of cherry tomato seedlings substrates grown in tested substrates.

Substrate	Germination (%)	GSI	MGT (days)	MGS (cm/days)	Root Length (cm)	Aerial part Length (cm)
Control	100 a	4.01 a	8.166 a	0.122 a	11.5 a	9.3 a
T1	100 a	2.23 d	14.44 e	0.069 e	10.1 a	6.6 b
T2	63 c	1.27 e	16.05 e	0.062 e	6.2 c	6.5 b
T3	71 c	1.51 e	15.13 e	0.066 e	4.6 c	4.1 c
T4	100 a	3.35 b	9.697 b	0.103b	8.2 b	4.3 c
T5	100 a	2.93 c	11.12 c	0.090c	8.9 b	5.0 c
T6	100 a	2.48 d	13.10 d	0.076 d	9.2 b	4.1 c
T7	95 a	1.99 d	15.21 e	0.065 e	9.1 b	6.5 b
T8	82 b	2.14 d	12.91 d	0.078 d	7.9 b	5.3 c

GSI: Germination Speed Index; MGT: Mean Germination Time; MGS: Mean Germination Speed. Averages followed by distinct numbers on rows differ from each other at a significance level of 5% for the Scott-Knott test. An absence of letters indicates that a test did not present statistical differences.

Within the context of the premises of a circular economy that involves nutritional conditioners (NCs) based on residues from the palm oil industry, from fungiculture (SMS) productive chains, from palm oil culture (lignocellulosic residues) and olericulture (cherry tomatoes), the addition of NCs based on such residues in the formulated substrate T1 (substrate colonized with *A. bisporus*) appear to favor plant physicochemical properties and/or biological activities that promote greater development of roots and aerial parts of the plants, appropriate for standardizing efficient production of tomato seedlings.

Results showed that the addition of NCs improved the performance of the substrates colonized with *A. bisporus* to similar or even superior levels to those obtained with the control for the production of tomato seedlings (Table 4). Treatments T10 and T12 stood out from the others, with results equal to the control for all parameters evaluated, with the exception of the length of aerial plant parts, for which T10 and T12 showed results superior to the control. Therefore, these results show that the by-products of the palm oil industry are excellent NCs for substrates employed in tomato seedling production. T9 also showed promising results for all evaluated parameters, with the exception of germination percentage, which was lower than observed in the control. In comparison with T10 and T12, treatment T9 resulted in plants with shorter aerial length, although this was similar to the control. The difference between formulation T9 and formulations T10 and T12 was the absence of palm oil fruit bunch fiber in the former, indicating a beneficial effect provided by this fiber.

**Table 4:** Germination and physical parameters (root and aerial part length) of substrates for the production of cherry tomato seedlings using substrate colonized with *A. bisporus* (T1) and nutritional conditioners.

Treatments	Germination (%)	GSI	MGT (days)	MGS (days)	Root Length (cm)	Aerial part Length (cm)
**Control**	97 a	5,04 a	6,06 a	0,16	9,9 a	8,4 b
**T9**	74 b	3,76 a	4,76 a	0,22	5,9b	8,1 b
**T10**	80 b	4,19 a	4,94 a	0,20	9,0 a	14,6 a
**T11**	31 c	1,38 b	2,37b	0,55	4,7b	5,5b
**T12**	91 a	4,66 a	5,89 a	0,17	8,5 a	11,9 a

GSI: Germination Speed Index; MGT: Mean Germination Time; MGS: Mean Germination Speed. Averages followed by distinct numbers on rows differ from each other on a significance level of 5% for the Scott-Knott test. Absence of letters means that the test did not present statistical differences. CO: Control (Commercial substrate); T9: 50% Substrate colonized with *A. bisporus* + 15% Palm kernel cake + 15% Palm oil + 20% Palm oil fiber; T10: 50% Substrate colonized with *A. bisporus* + 15% Palm kernel cake + 15% Palm oil + 20% Palm oil fruit bunch; T11: 50% Substrate colonized with *A. bisporus* + 25% Palm kernel cake + 25% Palm oil ash; T12: 50% Substrate colonized with *A. bisporus* + 15% Palm kernel cake + 15% Palm oil ash + 10% Palm oil fiber + 10% Palm oil fruit bunch.

Curiously, treatment T11 presented the worst results for all parameters evaluated, with the exception of aerial part length, for which there was no significant difference in relation to the control. The substrate from T11 showed higher compaction compared to the other substrates, which may have negatively interfered with plant germination and development. Moreover, this substrate received a higher proportion of ash in relation to the others, which may also have raised the salinity of the substrate to a higher level than that recommended for seedling production substrates.

**Fig. 3:**
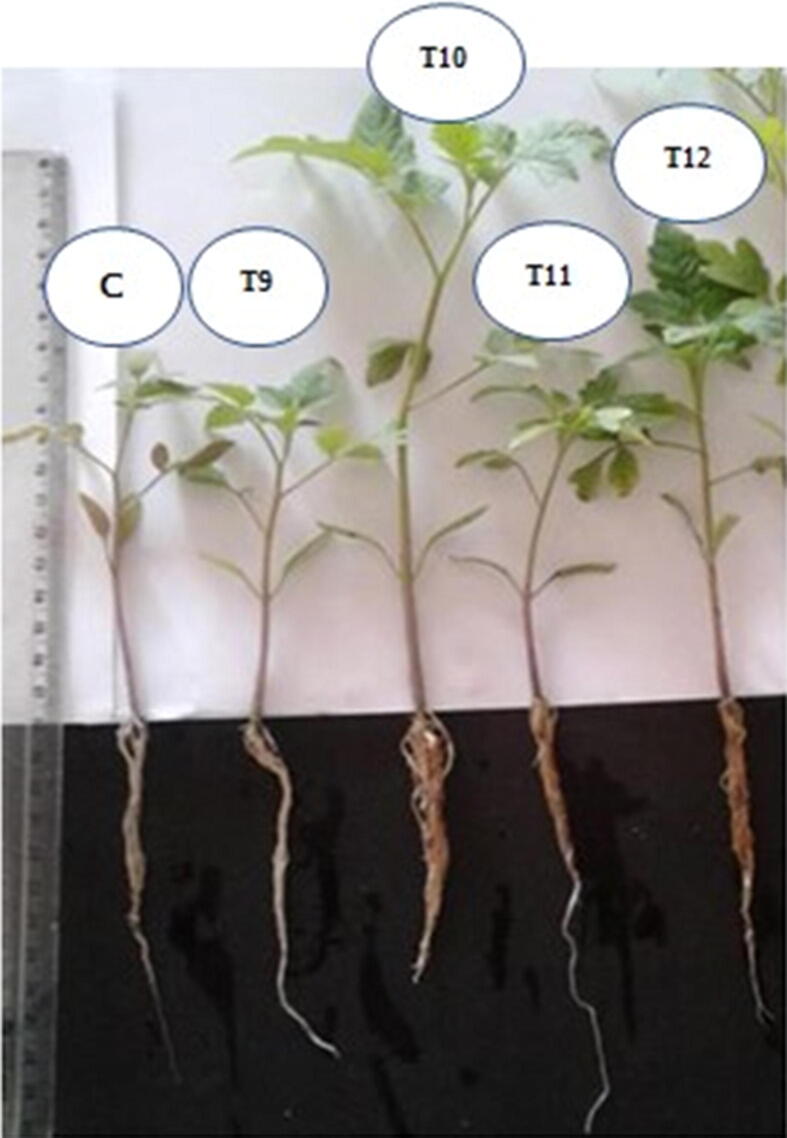
Tomato plant samples for evaluation of root growth and aerial part parameters after 30 days cultivation. CO: Control (Commercial substrate); T9: 50% Substrate colonized with *A. bisporus* + 15% Palm kernel cake + 15% Palm oil + 20% Palm oil fiber; T10: 50% Substrate colonized with *A. bisporus* + 15% Palm kernel cake + 15% Palm oil + 20% Palm oil fruit bunch; T11: 50% Substrate colonized with *A. bisporus* + 25% Palm kernel cake + 25% Palm oil ash; T12: 50% Substrate colonized with *A. bisporus* + 15% Palm kernel cake + 15% Palm oil ash + 10% Palm oil fiber + 10% Palm oil fruit bunch.

Results of quantitative parameters from seedlings produced with the substrate T10 are shown in Table 4. Although only enabling a qualitative analysis, which was not statistically significant, a superiority in seedlings produced in the T10 substrate was observed in terms of root growth and aerial part, which were well developed and ready for transplantation (Fig. 3).

In order to improve the physicochemical parameters of the formulation T10 selected from experiment 2, the addition of vermiculite was tested in proportions of 25% or 50% of the total substrate. Although it was expected that the addition of vermiculite would increase the substrate’s porosity and water retention capacity, this did not result in improved germination rates or seedling quality (Table 6). For germination percentage and GSI, the addition of vermiculite had a negative effect, with values below the control.

### 3.2 Analysis of mineralogical composition

Analysis of primarily essential macronutrients in T10 (50% substrate colonized with *A. bisporus* + 15% Palm kernel cake + 15% Palm oil + 20% Palm oil fruit bunch) substrate showed values of P and K higher than those observed in the control treatment (commercial substrate), while the values of N were identical in both substrates (Table 5).

**Table 5:** Comparison of the chemical characteristics of the most promising substrate for production of cherry tomato seedlings (T10) versus control (commercial substrate).

Analysis	Sample (%)	
	**Control (Commercial Substrate)**	**T10**
pH CaCl_2_ (0.01 M)	5.10	6.25
CEC (mmol L^-1^)	600.00	394.00
Humidity (60–65 °C)	10.20	7.46
Organic Carbon	5.63	5.44
Total Organic Matter	9.72	9.38
Total Nitrogen	0.70	0.70
Total Phosphorus (P_2_O_5_)	1.60	5.02
Potassium (K_2_O)	0.60	1.20
Zinc	0.001	0.010
Manganese	0.020	0.020
Calcium	0.75	2.27
Magnesium	4.31	0.47
Sulfur	0.01	0.55
Boron	0.001	0.010
Copper	0.002	0.010

CEC: cation-exchange capacity.

**Table 6:** Substrate formulations for the production of cherry tomato seedlings from experiment 3 and respective parameters for germination and seedlings quality.

Treatments	G (%)	GSI	MGT (days)	MGS (days)	DW	WW	% H_2_O	Root length (cm)	Aerial part length (cm)
**Co**	93 a	3.8 a	6.6	0.15	0.030 a	0.31 a	89	10 a	5 a
**T13**	71 b	2.4 c	6.0	0.16	0.027 a	0.38 a	92	7.3 ab	4 a
**T14**	90 a	3.2 b	7.4	0.13	0.026 a	0.35 a	92	9.0 ab	4 a

GSI: Germination Speed Index; MGT: Mean Germination Time; MGS: Mean Germination Speed; DW: Dry weight of tomato plants; WW: Wet weight of tomato plants; % H_2_O: The difference between dry weight and wet weight; CO: Control (Commercial substrate); T13 = T10 + Vermiculite 25%; T14 = T10 + 50% vermiculite. Averages followed by distinct numbers on rows differ from each other on a significance level of 5% for the Scott-Knott test. Absence of letters means that the test did not present statistical differences.

**Fig. 4:**
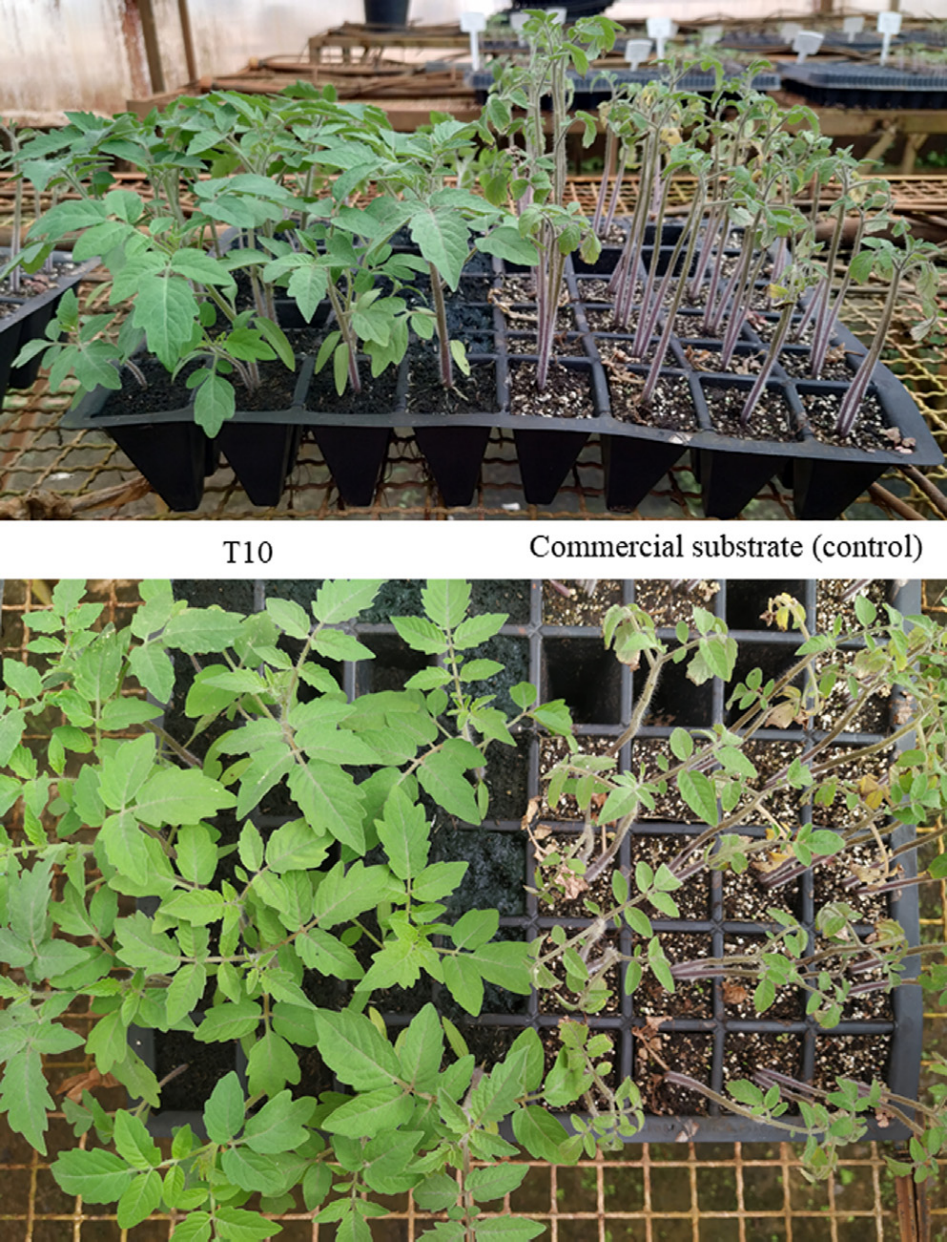
Cherry tomato seedlings 60 days after sowing on formulation T10 in comparison with the commercial substrate.

In addition to adequate mineral concentrations, results indicated that T10 substrate presented a composition that allows aeration and maintenance of humidity, thus favoring tomato seed germination and development of seedlings for a longer period. Another interesting result was the viability of tomato seedlings after 60 days of sowing. The substrate composed of T10 was able to maintain the vigor of tomato seedlings for a longer time compared to the commercial substrate, as shown in Fig. 4.

### 3.3 Co-cultivation of tomatoes and mushrooms

Given the literature that shows the potential of integrating mushroom production with agricultural production and the positive results obtained in this study with T10 substrate for production of tomato seedlings, a system of co-cultivation of tomato and mushrooms (*P. ostreatus*) was examined in a controlled environment setup (acclimatized greenhouse). The system was designed to enable both mushroom harvesting after two production periods, together with a soil microenvironment appropriate for efficient cherry tomato production (Fig. 1). In this system, soil-substrate enriched with nutrients met both the physiological demands of the plant and provided nutrients (N and others) for the fungal substrate, promoting the physiological processes of mushroom fruiting. It should be noted that both the tomatoes and mushrooms were produced in a controlled environment that was not 100% ideal for either organism but offering intermediate growth conditions to favor the co-cultivation.

**Fig. 5:**
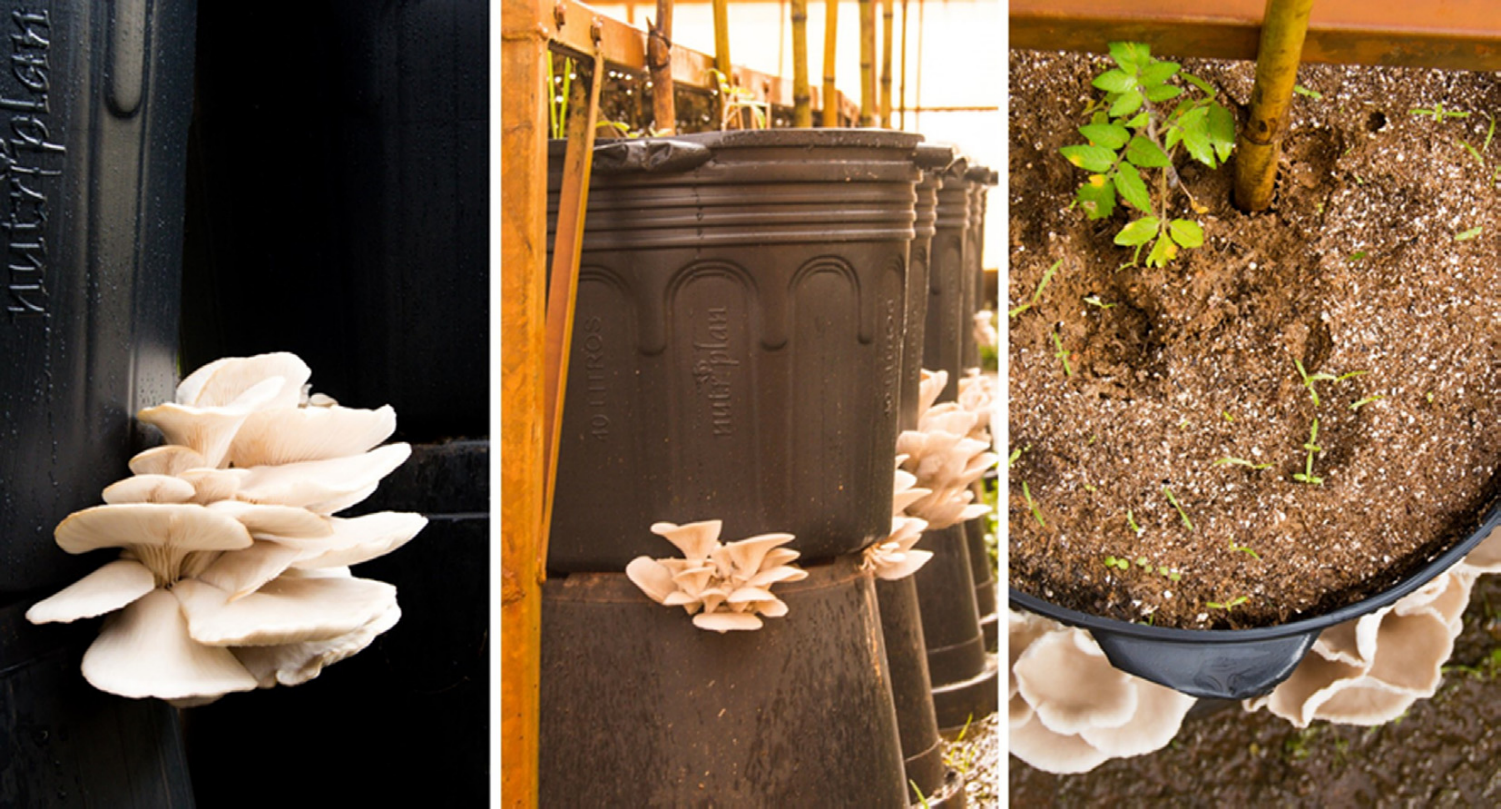
Illustration showing mushroom production during the first 10 days of co-cultivation, with cherry tomato plants in the initial phase of seedling growth.

Five different treatments were tested, consisting of various soil-substrate combinations for the upper layer (Table 2) and SMS from *P. ostreatus* in the middle layer for all treatments. Mushrooms began to appear 10 days after the beginning of the co-cultivation (Fig. 5) and continued to fruit until the end of the experiment (120 days).

**Fig. 6:**
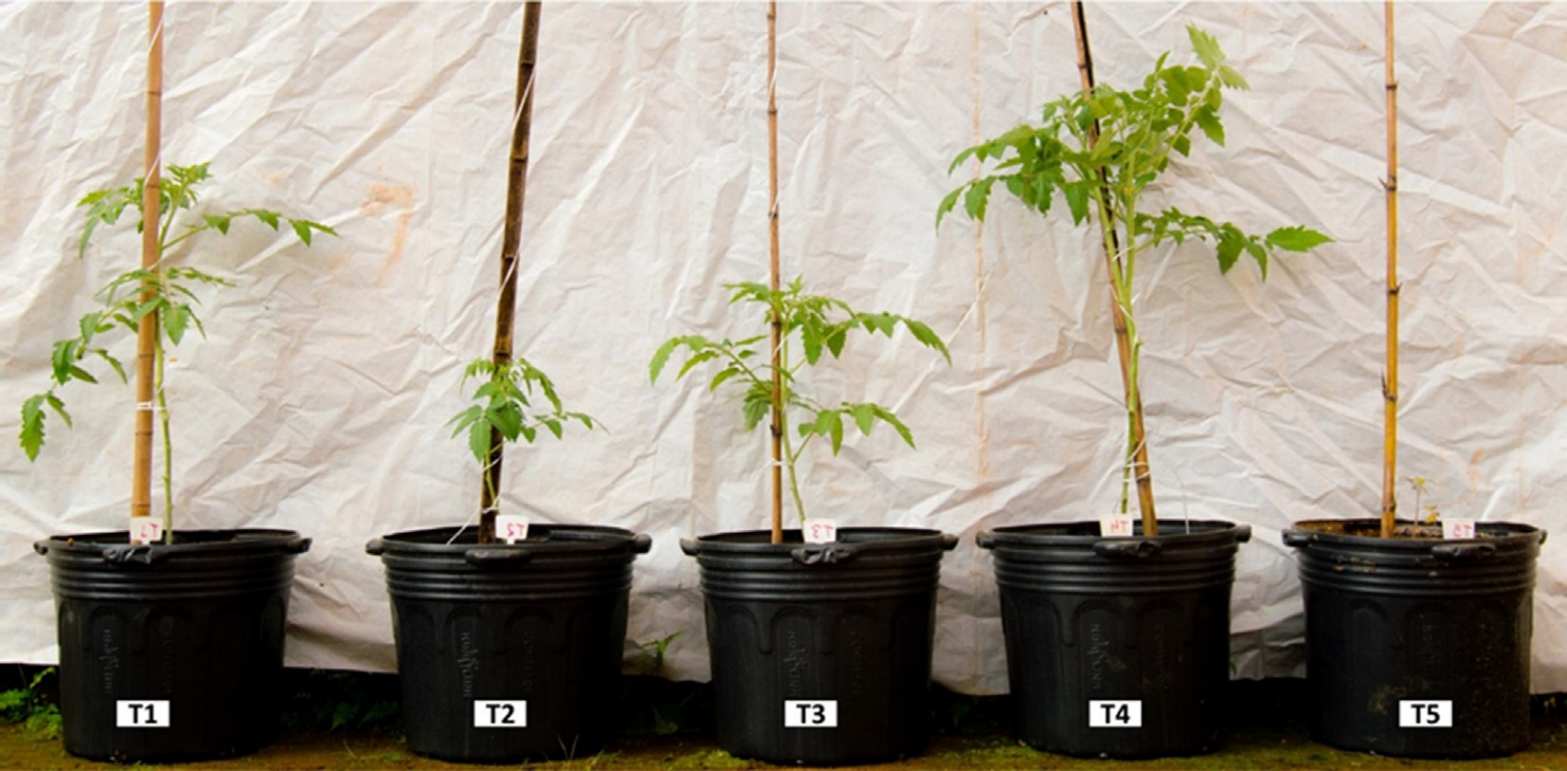
Cherry tomato plant growth after 30 days of co-cultivation with *P. ostreatus*. Treatments differed with regard to the content of the upper layer only. T1: Soil + Superficial fertilization with commercial fertilizer (5.7 g Ammonium Sulfate, 19 g MAP (Monoammonium Phosphate), 7 g KCl (Potassium Chloride); T2: 50% soil + 25% substrate colonized with *A. bisporus* + 12.5% Palm kernel cake + 12.5% Palm fruit bunch fiber; T3: 50% soil + 50% substrate for the cultivation of *A. bisporus* (same used in Step 2, but not inoculated); T4: 50% soil + 50% substrate colonized with *A. bisporus*; T5: 50% soil + 50% SMS *P. ostreatus*.

After 30 days co-cultivation, noticeable differences were apparent in tomato plant development ascorss the treatments (Fig. 6), with T4 (soil + substrate colonized with *A. bisporus*) the most noticeable in terms of growth promotion. This treatment was also the first to result in flowering, approximately 50 days after seedling transplanting.

**Fig. 7:**
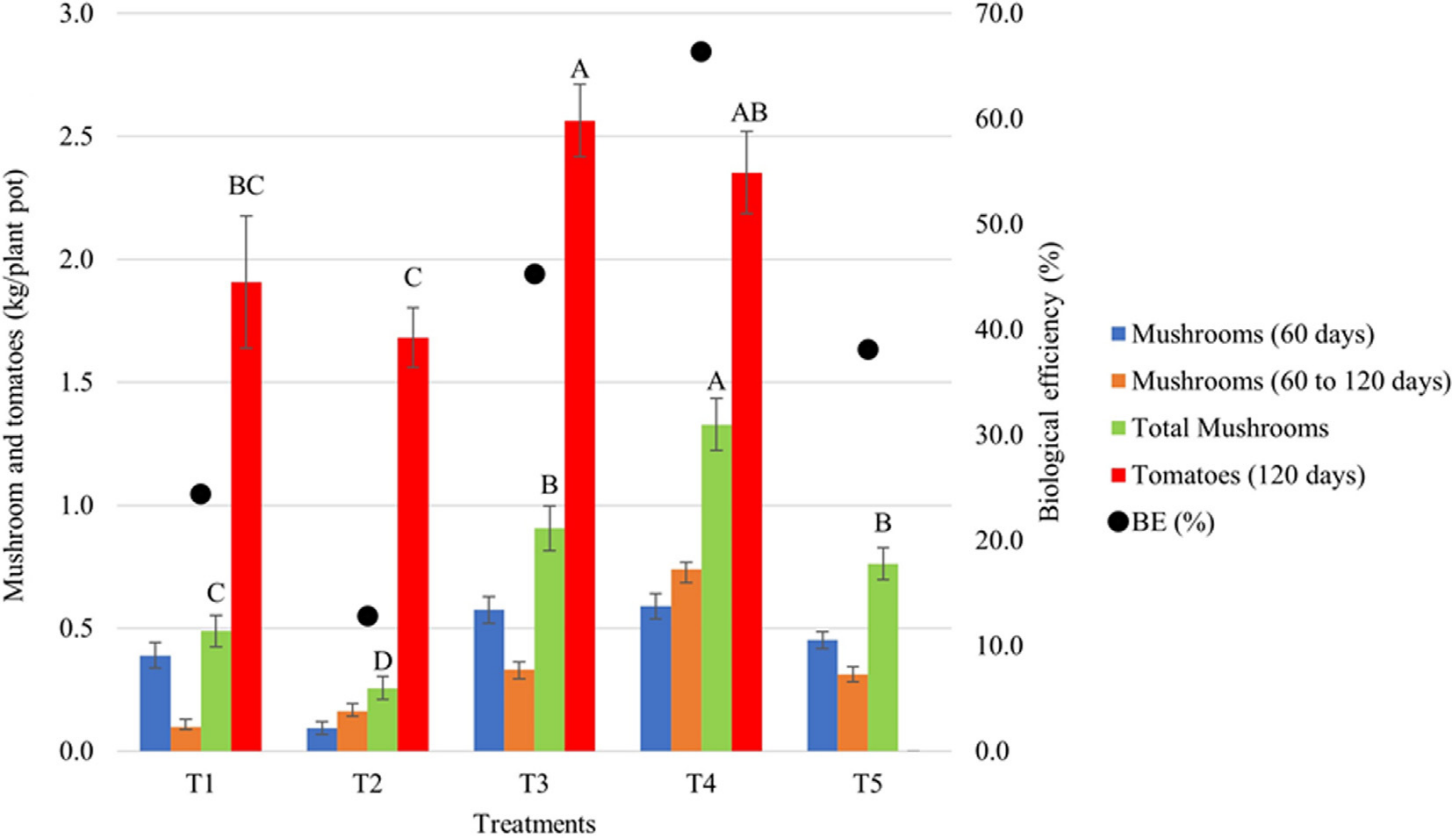
Production of fresh mushrooms (*Pleurotus ostreatus*) and cherry tomatoes in the integrated production system at 60 and 120 days. Treatments differed in terms of the content of the upper layer only. T1: Soil + Superficial fertilization with commercial fertilizer; T2: 50% soil + 25% substrate colonized with *A. bisporus* + 12.5% Palm kernel cake + 12.5% Palm oil fruit bunch fiber; T3: 50% soil + 50% substrate for the cultivation of *A. bisporus* (same used in experiment 2, but not inoculated); T4: 50% soil + 50% substrate colonized with *A. bisporus*; T5: 50% soil + 50% SMS P. *ostreatus*. BE – Biological efficiency. In order to compare both mushroom and tomato yield, capital letters were used for the comparison of the same groups across different treatments (bars). Different letters differ from each other at a significance level of 5% for the Tukey test.

Results obtained following co-cultivation (Fig. 7) were evaluated in terms of production (yield) of fresh mushrooms (Kg fresh mushrooms/pot) before and after nutritional supplementation, biological efficiency (BE = g fresh mushrooms/g of dry substrate), and tomato production (Kg per plant over a 60 days of harvest). Tomato yields were highest at T3 and T4, with 2.6 and 2.4 Kg/pot, respectively.

Mushroom yield over the 120 days of co-cultivation in T4 was significantly higher than observed in other treatment (1.3 Kg/pot), and also presented the best biological efficiency (66.4%). Mushroom production after plant supplementation at 60 days displayed better results only with T4, indicating that supplementation with N interfered positively with the growth of *P. ostreatus*. For T1, T2, T3 and T5 biological efficiencies 24.4%, 12.8%, 45.3%, and 38.1%, were observed, respectively.

## 4 Discussion

A number of studies have reported that SMS contains high levels of organic matter, carbohydrates, proteins, lipids, nitrogen, phosphorus, potassium and other nutrients, with such characteristics suitable in applied in agricultural systems (Jordan et al., 2008, Medina et al., 2012, Roy et al., 2015). The benefits of SMS as an organic fertilizer and soil conditioner have also been reported (Courtney and Mullen, 2008, Hackett, 2015). In addition to organic matter, SMS also have a population of microorganisms, in addition to the original fungus, which are beneficial for plant development, favoring competition with natural pathogens such as pathogenic fungi and nematodes, as demonstrated in the work of de Moraes et al., (2020).

The use of SMS from mushroom production has demonstrated the effectiveness of these materials in agriculture as substitutes for substrate components in the production of seedlings of different plant cultivars. Zhang et al. (2012) described SMS as a suitable co-product for the production of vegetable seedlings, using this substrate for greenhouse production of cucumber and tomato seedlings. According to the authors, SMS served as a potential substitute for peat, providing better physicochemical properties that enabled good growth and development of cucumber seedlings. Porto et al. (2018) observed a positive effect on germination time when SMS from *A. bisporus* was added to substrate for the cultivation of bell pepper. This effect may be due to the rapid release of a large number of ions which can be easily absorbed by the plant, resulting in an increase in dry matter in a short period of time (Rashid et al., 2018a, Rashid et al., 2018b).

A number of benefits of SMS in agriculture have been described (Hanafi et al., 2018), for example in the integration of fungiculture (SMS or colonized substrates) with the development of products such as biofertilizers, using waste from two production chains. The use of SMS has been indicated in circular economy models (Grimm and Wösten, 2018), being applied as a substrate for substitution of peat usage in plant production. In several countries, the use of natural peat (extraction from spring environments) is now prohibited or restricted (Paula et al., 2017, Yau and Murphy, 2000, Zhang et al., 2012), given the negative environmental impact of it́s extraction and resulting soil structure disturbance (Moxey and Moran, 2014).

Another important aspect of this type of substrate is the potential positive interaction between plant and microorganisms present. In addition to the metabolites produced by *A. bisporus* and its own cell wall constituents (chitin and β-glucans), these substrates also present a rich microbiota which can influence the rhizosphere region (de Moraes et al., 2020), favoring the establishment of a microbiota beneficial for tomato seedling production. Tall and Meyling (2018) suggested that the microorganism *Beauveria bassiana* is able to interact beneficially with corn plants depending on the nutritional availability of the substrate. The authors described that the interaction could vary from antagonist to mutualist and conclude that the plant-fungal relationship is therefore complex, contradictory, and context dependent. Nonetheless, as described herein, the inclusion of SMS from *A. bisporus* mushrooms in substrates for cultivation or seedling production has shown positive results. The colonized substrate of *A. bisporus*, also presents good qualities as a substrate for tomato. In the presente experiment, the addition of a nutritional conditioner based on palm residue improves the quality of the colonized substrate of *A. bisporus* for tomato seedlings.

For plant development, in addition to interactions with microorganisms, the substrate needs to have an adequate balance of nutrients, which were mostly found in the T10 substrate of this work such as Phosphorus and Potassium, both favoring the development of the plant (Brito et al., 2018). Although phosphorus is an essential element for plant growth, a is normally abundantly present in soil, it cannot be assimilated directly by plants. Vassilev et al. (2006) showed the potential of using microorganisms in the processes of phosphorus solubilization in fermentative systems as an alternative to fertilizer production, since organic acid producing microorganisms are able to solubilize phosphates of aluminum, iron and calcium. According to Helm et al. (2009), among other nutrients essential to the quality development of plants are calcium and magnesium, where the first stimulates the absorption of other ions to maintain the structure and normal function of cell membranes, and the later acts on the composition of chlorophyll, proto-chlorophyll, pectin and phytin. Micronutrients, such as copper, iron, manganese or zinc, are important as cofactors in the activation of several plant enzymes, in the formation of chlorophyll and indoleacetic acid, nitrogen absorption, and in enzymatic processes in general for plant growth (Brito et al., 2018). Substrate pH is also important in seedling production, as it influences the availability of nutrients and physiological balance of the plant. Shibata and Demiate (2003) described an ideal pH between 6.0 and 7.0 for nutrient availability.

Vermiculite is a common component in several substrate formulations for seedlings production, given its desirable physicochemical characteristics. However, the results obtained in this study show that the addition of vermiculite is not always necessary. A potential explanation for this may be that the *A. bisporus* substrate already provides the same benefits as vermiculite, although this may vary according to the components employed in the formulation of the *A. bisporus* mushroom substrate.

The tomato production yield in the co-culture conditions is in accord with literature that describes alternative substrates such as vermicompost with coconut fibers and rice husk ash (Truong et al., 2018), or mixtures of vermicompost, vermiculite and cattle manure (Costa et al., 2018). Most tomato harvests observed in these previous studies occurred in up to 80 days (Oliveira et al., 2020), in contrast to this study, where an earlier harvest was observed at only 60 days in average.

SMS of *Agaricus* species has proven to be a good substrate component for fertigation of vegetables such as tomato, lettuce and cucumber (Stoknes et al., 2019). This was also observed in the current work, with the treatment containing substrate colonized with *A. bisporus* (T4) showing better results in terms of tomato growth and productivity, when compared to the control and T5, which received SMS of *Pleurotus* without the addition of any nutritional conditioners (Fig. 7). Other crops such as lettuce and cucumber have also benefited from the use of biomass from fungiculture, such as in the form of organic fertilizers (Elsakhawy and tawfik, 2019), demonstrating that there is potential in the integration of production chains. In this work, it became evident that in addition to the production of substrates for plant seedlings, it was also possible to produce mushrooms (*P. ostreatus*) and tomatoes in the same environment, further strengthening the principle of a circular economy.

The action of degradation of plant matter by fungi is widely studied and they also develop important functions in symbiotic interactions with plants. Much has been discussed about the importance of endophytic fungi for the development of plants and vice versa (Kothe and Turnau, 2018). Basidiomycetes known as white-rot fungi are highly related to decomposition processes due to their lignocellulolytic enzymes (Sudarson et al., 2014), but little is discussed about the beneficial interactions with living plants. Mushroom, such as those from Pleurotus Genera have been demonstrate great potential against nematodes which can benefit the plant development (Elkhateeb, Daba and Soliman, 2021). The interaction between plants and fungi is a sophisticated strategy in which both benefit using a chemical language with the production and secretion of several biomolecules, including phytohormones that can modulate plant development (Rashid et al., 2018a, Rashid et al., 2018b). Understanding this relationship will allow for greater food productivity in an efficient and sustainable manner.

The cultivation of different mushroom species offers potential in different agricultural production systems through the use of SMS as raw material for animal food (birds, fish, and larvae), biofertilizers, bionematicide, biofungicide and others. Industrialized nations could potentially include this in strategies to achieve self-sustaining environments, while for developing nations the main incentive would be to promote mushroom cultivation in circular food chains, likely achieving food security and better public health. Smallholder farmers could also profit through mushroom cultivation as an additional source of income and food (Grimm et al., 2020). This circular economy model that integrates fungiculture with other sectors offers considerable potential for implementation in different regions of Brazil, given the availability of green biomasses for mushroom production, as well as the need to generate alternative sources of food and income for smallholder farms. The oil palm industry and the oil processing agroindustry generate many lignocellulosic residues, which can be a source of raw material for the production of mushrooms, which can then provide generated SMS applicable as a fertilizer for different crops. Grimm et al. (2020) reviewed potential approaches to integrate mushroom production with other food generating chains, reporting that the application of 100 tons of SMS from *A. bisporus* per hectare resulted in a 59% increase in grain production as a result of higher levels of soil phosphorus, potassium, nitrogen and organic matter.

Here we show, for the first time, how smallholder farmers may benefit from the co-production of mushrooms and olericulture in regions that both require improved living conditions and that generate large amounts of agroindustrial by-products, such as those from the oil palm industry. In this way, results presented herein provide a basis for the successful implementation of a circular economy model through the integration of mushroom and cherry tomato production.

## 5 Conclusion

This work shows that it is possible to integrate productive chains in a sustainable system such as fungiculture, olericulture, and palm oil culture through the use of spent mushroom substrate (SMS) or colonized substrates from *A. bisporus* supplemented with residues from the palm oil agroindustry for the production of cherry tomato seedlings. Furthermore, an integrated and sustainable system of co-production of tomato and mushrooms is proposed that is applicable to smallholder farmers.

## Funding

This study was funded by 10.13039/501100004809FINEP, 10.13039/501100003593CNPq and 10.13039/501100005286FAPEMAT

## Consent to participate

Not applicable.

## Consent for publication

Not applicable.

## Availability of data and material

All data generated or analyzed during this study are included in this published article.

## Code availability

Not applicable.

## CRediT authorship contribution statement

**Vandinelma Oliveira Vieira:** Conceptualization, Methodology, Investigation, Writing – original draft. **Aparecido Almeida Conceição:** Conceptualization, Investigation, Writing – original draft, Writing – review & editing. **Joice Raisa Barbosa Cunha:** Investigation. **Antony Enis Virginio Machado:** Conceptualization, Investigation. **Euziclei Gonzaga de Almeida:** Writing – review & editing, Funding acquisition, Supervision. **Eustáquio Souza Dias:** Writing – review & editing, Resources, Supervision. **Lucas Magalhães Alcantara:** Writing – original draft, Writing – review & editing. **Robert Neil Gerard Miller:** Writing – review & editing. **Félix Gonçalves de Siqueira:** Conceptualization, Methodology, Investigation, Writing – review & editing, Funding acquisition, Resources, Supervision.

## Declaration of Competing Interest

The authors declare that they have no known competing financial interests or personal relationships that could have appeared to influence the work reported in this paper.
